# Standard errors and confidence intervals in within-subjects designs: Generalizing Loftus and Masson (1994) and avoiding the biases of alternative accounts

**DOI:** 10.3758/s13423-012-0230-1

**Published:** 2012-03-23

**Authors:** Volker H. Franz, Geoffrey R. Loftus

**Affiliations:** 1 Universität Hamburg, von Melle Park 5, 20146 Hamburg, Germany; 2 University of Washington, Seattle, Washington USA

**Keywords:** Statistics, Statistical inference, Confidence intervals, Repeated measures

## Abstract

Repeated measures designs are common in experimental psychology. Because of the correlational structure in these designs, the calculation and interpretation of confidence intervals is nontrivial. One solution was provided by Loftus and Masson (*Psychonomic Bulletin & Review 1*:476–490, 1994). This solution, although widely adopted, has the limitation of implying same-size confidence intervals for all factor levels, and therefore does not allow for the assessment of variance homogeneity assumptions (i.e., the circularity assumption, which is crucial for the repeated measures ANOVA). This limitation and the method’s perceived complexity have sometimes led scientists to use a simplified variant, based on a per-subject normalization of the data (Bakeman & McArthur, *Behavior Research Methods, Instruments, & Computers 28*:584–589, 1996; Cousineau, *Tutorials in Quantitative Methods for Psychology 1*:42–45, 2005; Morey, *Tutorials in Quantitative Methods for Psychology 4*:61–64, 2008; Morrison & Weaver, *Behavior Research Methods, Instruments, & Computers 27*:52–56, 1995). We show that this normalization method leads to biased results and is uninformative with regard to circularity. Instead, we provide a simple, intuitive generalization of the Loftus and Masson method that allows for assessment of the circularity assumption.

Confidence intervals are important tools for data analysis. In psychology, confidence intervals are of two main sorts. In *between-subjects* designs, each subject is measured in only one condition, such that measurements across conditions are typically independent. In *within-subjects* (repeated measures) designs, each subject is measured in multiple conditions. This has the advantage of reducing variability caused by differences among the subjects. However, the correlational structures in the data cause difficulties in specifying confidence-interval size.

Figure 1a shows hypothetical data from Loftus and Masson (1994). Each curve depicts the performance of one subject in three exposure-duration conditions. Most subjects show a consistent pattern—better performance with longer exposure duration—which is reflected by a significant effect in repeated measures analysis of variance (ANOVA) [*F*(2, 18) = 43, *p* < .001].

**Fig. 1:**
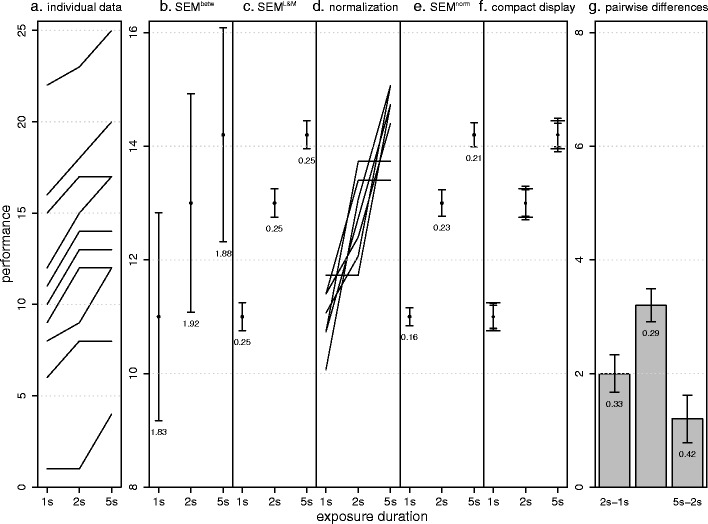
Hypothetical data of Loftus and Masson (1994). (**a**) Individual data: Each subject performs a task under three exposure durations (1 s, 2 s, and 5 s). Although the subjects vary in their overall performance, there is a clear within-subjects pattern: All subjects improve with longer exposure duration. (**b**) The between-subjects *SEM* ^betw^ values don’t reflect this within-subjects pattern, because the large between-subjects variability hides the within-subjects variability. (**c**) *SEM* ^L&M^, as calculated by the Loftus and Masson method, adequately reflects the within-subjects pattern. (**d**) The normalization method: First, the data are normalized (**e**). Second, traditional *SEM*s are calculated across the normalized values, resulting in *SEM* ^norm^. (**f**) Our suggestion for a compact display of the data. Error bars with long crossbars correspond to *SEM* ^L&M^, and error bars with short crossbars to *SEM* ^pairedDiff^ (scaled by the factor 1/√2; see main text). The fact that the *SEM* ^pairedDiff^ values are almost equal to those of *SEM* ^L&M^ indicates that there is no serious violation of circularity. (**g**) Pairwise differences between all conditions and the corresponding *SEM* ^pairedDiff^s. Error bars depict ±1 *SEM*s as calculated by the different methods. Numbers below the error bars are the numerical values of the *SEM*s

However, this within-subjects effect is not reflected by traditional standard errors of the mean (*SEM*; Fig. 1b), as calculated with the formula.

$$ SEM_j^{{betw}} = \sqrt {{\frac{1}{{n(n - 1)}}\sum\limits_{{i = 1}}^n {\,\,{{\left( {{y_{{ij}}} - \overline {{y_{{.j}}}} } \right)}^2}} }} $$

where *SEM*_*j*_^betw^ is the *SEM* in condition *j*, *n* the number of subjects, *y*_*ij*_ the dependent variable (*DV*) for subject *i* in condition *j*, and $\overline {{y_{{.j}}}}$ the mean *DV* across subjects in condition *j*.

The discrepancy occurs because the *SEM* ^betw^ includes both the subject-by-condition interaction variance—the denominator of the ANOVA’s *F* ratio—and in addition the between-subjects variance, which is irrelevant in the *F* ratio. In our example, subjects show highly variable overall performance, which hides the consistent pattern of within-subject effects. This is common: The between-subjects variability is typically larger than the subject-by-condition interaction variability. Therefore, the *SEM* ^betw^ is inappropriate for assessing within-subjects effects. Before discussing solutions to this shortcoming, we will offer some general comments about error bars.

## Error bars

Error bars reflect measurement uncertainty and can have different meanings. For example, they can correspond to *SEM*s, standard deviations, confidence intervals, or the more recently proposed inferential confidence intervals (Goldstein & Healy, 1995; Tryon, 2001). Each of these statistics stresses one aspect of the data, and each has its virtues. For example, standard deviations might be the first choice in a clinical context where the focus is on a single subject’s performance. In experimental psychology, the most-used statistic is the *SEM*. For simplicity, we will therefore focus on the *SEM*, although all of our results can be expressed in terms of any related statistic.

To better understand the *SEM*, it is helpful to recapitulate two simple “rules of eye” for the interpretation of *SEM*s. The rules, which we will call the *2-* and *3-SEM rules*, respectively, are equivalent to Cumming and Finch’s (2005) Rules 6 and 7. First, if a single mean (based on *n* ≥ 10 measurements) is further from a theoretical value (typically zero) than ~2 *SEM*s, this mean is significantly different (at *α* = .05) from the theoretical value. Second, if two means (both based on *n* ≥ 10 measurements) in a between-subjects design with approximately equal *SEM*s are further apart than ~3 *SEM*s, these means are significantly different from one another (at *α* = .05).^1^

## Loftus and Masson (1994) method

Loftus and Masson (1994) offered a solution to the problem that *SEM* ^betw^ hides within-subject effects (Fig. 1c). The *SEM* ^L&M^ is based on the pooled error term of the repeated measures ANOVA and constructed such that the 3-*SEM* rule can be applied when interpreting differences between means. This central feature makes the *SEM* ^L&M^ in a repeated measures design behave analogously to the *SEM* ^betw^ in a between-subjects design.^2^

## Normalization method

Although widely accepted, Loftus and Masson’s (1994) method has two limitations: (a) By using the pooled error term, the method assumes *circularity*, which to a repeated measures design is what the homogeneity of variance (HOV) is to a between-subjects design. Consequently, all *SEM* ^L&M^s are of equal size. This is different from between-subjects designs, in which the relative sizes of the values of *SEM* ^betw^ allow for judgments of the HOV assumption. (b) The formulas by Loftus and Masson (1994) are sometimes perceived as unnecessarily complex (Bakeman & McArthur, 1996).

Therefore, Morrison and Weaver (1995), Bakeman and McArthur (1996), Cousineau (2005), and Morey (2008) suggested a simplified method that we call the *normalization method*. It is based on an illustration of the relationship between within- and between-subjects variances used by Loftus and Masson (1994).^3^ Proponents of the normalization method argue that it is simple and allows for judgment of the assumption of circularity.

The normalization method consists of two steps. First, the data are normalized (Fig. 1d). That is, the overall performance levels for all subjects are equated without changing the pattern of within-subjects effects. Normalized scores are calculated as

$$ {w_{{ij}}} = {y_{{ij}}} - \left( {\overline {{y_{{i.}}}} - \overline {{y_{{..}}}} } \right) $$

where *i* and *j* index the subject and factor levels; *w*_*ij*_ and *y*_*ij*_ represent normalized and raw scores, respectively; $\overline {{y_{{i.}}}}$ is the mean score for subject *i*, averaged across all conditions; and $\overline {{y_{{..}}}}$ is the grand mean of all scores. Second, the normalized scores *w*_*ij*_ are treated as if they were from a between-subjects design. The rationale is that the irrelevant between-subjects differences are removed, such that now standard computations and the traditional *SEM* formula can be used on the normalized scores:

$$ SEM_j^{{norm}} = \sqrt {{\frac{1}{{n(n - 1)}}\sum\limits_{{i = 1}}^n {{{\left( {{w_{{ij}}} - \overline {{w_{{.j}}}} } \right)}^2}} }} $$

with *SEM*_*j*_^norm^ being the *SEM* ^norm^ in condition *j* and *n* the number of subjects. The resulting *SEM* ^norm^s are shown in Fig. 1e.

The normalization method seems appealing in its simplicity. All that is required is to normalize the within-subjects data, and then standard methods from between-subjects designs can be used. However, this method underestimates the *SEM*s and does not allow for an assessment of circularity.

## Problem 1 of the normalization method: SEMs are too small

Figures. 1c and 1e illustrate this problem: all *SEM* ^norm^ values are smaller than *SEM* ^L&M^. This is a systematic bias that occurs because the normalized data, although correlated, are treated as uncorrelated. Consequently, the pooled *SEM* ^norm^ underestimates the *SEM* ^L&M^ by a factor of $\sqrt {{\frac{{J - 1}}{J}}}$ (with *J* being the number of factor levels).^4^ Morey (2008) derived this relationship and also suggested that the *SEM* ^norm^ be corrected. However, this is not a complete solution, because the method still leads to an erroneous view of what circularity means.

## Circularity

Between-subjects ANOVA assumes HOV, and we can assess the plausibility of this assumption by judging whether the *SEM* ^betw^ values are of similar size. The corresponding assumption for repeated measures ANOVA is *circularity* (Huynh & Feldt, 1970; Rouanet & Lepine, 1970).

Consider the variance–covariance matrix **Σ** of a repeated measures design. Circularity is fulfilled if and only if an orthonormal matrix **M** exists that transforms **Σ** into a spherical matrix (i.e., with *λ* on the main diagonal and zero elsewhere), such that

$$ {\mathbf{M\Sigma M}}\prime = \lambda {\mathbf{I}} $$

where *λ* is a scalar and **I** is the identity matrix (cf. Winer, Brown, & Michels, 1991). Because of this relationship to sphericity, the circularity assumption is sometimes called the *sphericity assumption*.

We can reformulate circularity in a simple way: Circularity is fulfilled if and only if the variability of all pairwise differences between factor levels is constant (Huynh & Feldt, 1970; Rouanet & Lepine, 1970). Therefore, we can assess circularity by examining the variance of the difference between any two factor levels. Depicting the corresponding *SEM*, which we describe below, is an easy generalization of the Loftus and Masson (1994) method. Before describing this method, however, we show that the normalization method fails to provide correct information about circularity.

## Problem 2 of the normalization method: Erroneous evaluation of circularity

There are different reasons why the normalization method cannot provide a visual assessment of circularity. For example, testing for circularity requires evaluating the variability of all *J*(*J* − 1)/2 pairwise differences (*J* being the number of factor levels), while the normalization method yields only *J SEM* ^norm^ values to compare. Also, we can construct examples showing clear violations of circularity that are not revealed by the normalization method.

**Fig. 2:**
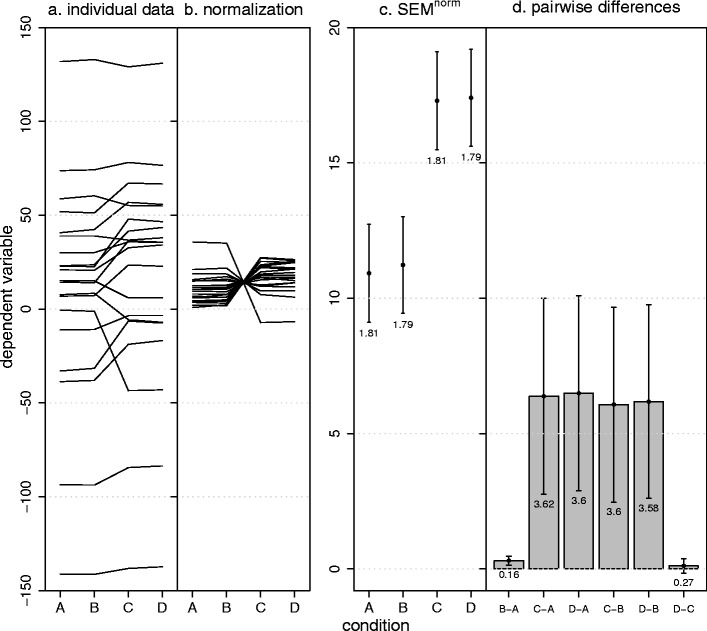
Example showing that the normalization method fails to detect serious violations of circularity. (**a**) Simulated data for a within-subjects factor with four levels. (**b**) The normalized data. (**c**) The normalization method leads to similar *SEM* ^norm^ values, thereby not indicating the violation of circularity. (**d**) Pairwise differences and the corresponding *SEM* ^pairedDiff^s indicate a large violation of circularity. Error bars depict ±1 *SEM*s as calculated by the different methods. Numbers below the error bars are the numerical values of the *SEM*s

Figure 2 shows such an example for one within-subjects factor with four levels. The pairwise differences (Fig. 2d) show small variability between levels A and B and levels C and D, but large variability between levels B and C. The normalization method does not indicate this large circularity violation (Fig. 2c). The reason can be seen in Fig. 2b: Normalization propagates the large B and C variability to conditions A and D. Because conditions A and B don’t add much variability themselves, the normalization method creates the wrong impression that circularity holds.

It is instructive to evaluate this example using standard measures of circularity. The Greenhouse–Geisser epsilon (Box, 1954a, 1954b; Greenhouse & Geisser, 1959) attains its lowest value at maximal violation [here, *ε*_min_ = 1/(*J* – 1) = .33], while a value of *ε*_max_ = 1 indicates perfect circularity. In our example, *ε* = .34, showing the strong violation of circularity (Huynh & Feldt’s, 1976, epsilon leads to the same value). The Mauchly (1940) test also indicates a significant violation of circularity (*W* = .0001, *p* < .001) and a repeated measures ANOVA yields a significant effect [*F*(3, 57) = 3, *p* = .036], but only if we—erroneously—assume circularity. If we recognize this violation of circularity and perform the Greenhouse–Geisser or Huynh–Feldt corrections, the effect is not significant (both *p*s = .1). A multivariate ANOVA (MANOVA) also leads to a nonsignificant effect [*F*(3, 17) = 1.89, *p* = .17]. In summary, our example shows that the normalization method can hide serious circularity violations. A plot of the *SEM* of the pairwise differences, on the other hand, clearly indicates the violation.

## A better approach: Picturing pairwise differences

As a simple and mathematically correct alternative to the normalization method, we suggest showing all pairwise differences between factor levels with the corresponding *SEM* (*SEM* ^pairedDiff^), as shown in Figs. 1g and 2d. To the degree that these values of *SEM* ^pairedDiff^ are variable, there is evidence for violation of circularity. Figure 1g shows that for the Loftus and Masson (1994) data, all *SEM* ^pairedDiff^s are similar, suggesting no serious circularity violation (which is consistent with standard indices: Greenhouse–Geisser *ε* = .845, Huynh–Feldt *ε* = 1, Mauchly test *W* = .817; *p =* .45).

The values of *SEM* ^pairedDiff^ are easy to compute, because only the traditional formulas for the *SEM* of the differences are needed. Consider the levels *k* and *l* of a repeated measure factor. We first calculate the pairwise differences for each subject *d*_*i*_ = *y*_*ik*_ – *y*_*il*_, then use the traditional formula to calculate the *SEM* of the mean difference:

$$ SEM_{{kl}}^{{pairedDiff}} = \sqrt {{\frac{1}{{n\left( {n - 1} \right)}}\sum\limits_{{i = 1}}^n {{{\left( {{d_i} - {{\overline d }_{.}}} \right)}^2}} }} $$

This approach is consistent with the Loftus and Masson (1994) method, because pooling the *SEM* ^pairedDiff^s results in $\frac{1}{{\sqrt {2} }}SE{M^{{{\text{L}}\& {\text{M}}}}}$ (Appendix A1). Therefore, we can use this relationship to calculate the *SEM* ^L&M^ without the inconvenience of extracting the relevant ANOVA error term from the output of a statistical program (another critique of the Loftus & Masson method: Cousineau, 2005; Morey, 2008).

## Picturing pairwise differences can supplement numeric methods

**Fig. 3:**
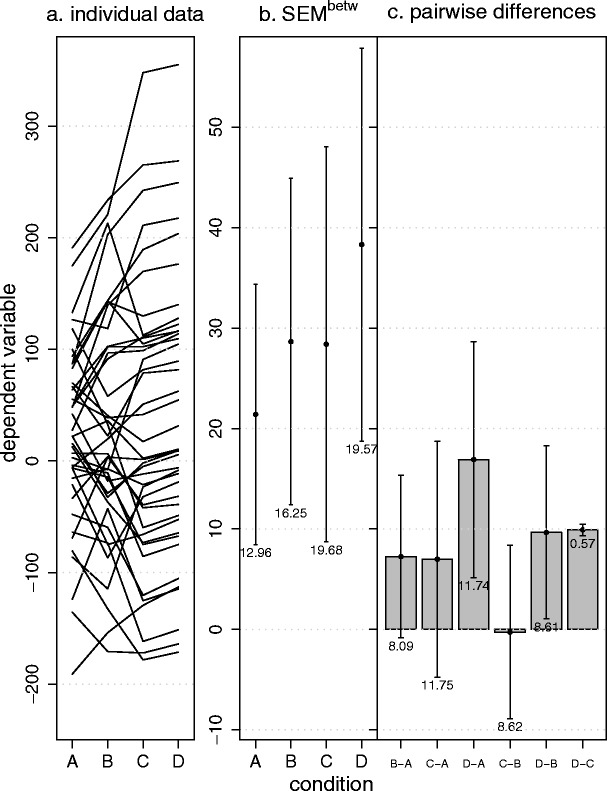
Example demonstrating the virtues of our approach. (**a**) Simulated data for a within-subjects factor with four levels. (**b**) Means and the corresponding *SEM* ^betw^ values. (**c**) The pairwise differences and corresponding *SEM* ^pairedDiff^s indicate a large violation of circularity. Error bars depict ±1 *SEM*s as calculated by the different methods. Numbers below the error bars are the numerical values of the *SEM*s

Figure 3 illustrates how evaluating *SEM* ^pairedDiff^ can lead to a surprising result, thereby showing the virtues of our approach. Repeated measures ANOVA shows for these data a clearly nonsignificant result, whether or not we correct for circularity violation [*F*(3, 117) = 1.2, *p* = .32; Greenhouse–Geisser *ε* = .50, *p* = .30; Huynh–Feldt *ε* = .51, *p* = .30]. We show that our method nevertheless detects a strong, significant effect and will guide the researcher to the (in this case) more appropriate multivariate methods.

Inspecting Fig. 3c for circularity violations shows that between conditions D and C there is a very small *SEM* ^pairedDiff^, indicating that the pairwise difference between these conditions has much less variability than all of the other pairwise differences. Applying the 2-*SEM* rule indicates that the corresponding difference differs significantly from zero, while no other differences are significant. This is also true, using the Bonferroni correction^5^ for multiple testing, as suggested by Maxwell and Delaney (2000).

In short, *SEM* ^pairedDiff^ indicates that there is a strong circularity violation and a strong effect. Univariate repeated measures ANOVA does not detect this effect, even when corrected for circularity violations. MANOVA, on the other hand, detects the effect [*F*(3, 37) = 98, *p* < .001] and is thereby consistent with the result of our approach.^6^

This example shows that the *SEM* ^pairedDiff^ conveys important information about the correlational structure of the data that can prompt the researcher to use more appropriate methods. No other method discussed in this article would have achieved this.

## Practical considerations when picturing pairwise differences

The example above shows that our approach can help the researcher during data analysis. When presenting data to a general readership, a more compact way of presenting the *SEM* ^pairedDiff^ might be needed, especially for factors with many levels [because the number of pairwise differences can become large; *J* factor levels will result in *J*(*J* – 1)/2 pairwise differences]. If a plot of pairwise differences would be overly tedious, one could (a) present the data as an upper triangular matrix, either in numerical form or as a color-coded heat map, or (b) present the *SEM* ^pairedDiff^ together with the *SEM* ^L&M^ in one single plot, as shown in Fig. 1f. In this plot, the error bars with short crossbars correspond to the *SEM* ^pairedDiff^ (scaled, see below), and the error bars with long crossbars correspond to the *SEM* ^L&M^. The plot gives a correct impression of circularity by means of the scaled *SEM* ^pairedDiff^s (if circularity holds, all scaled *SEM* ^pairedDiff^s will be similar to *SEM* ^L&M^) and allows for application of the 3-*SEM* rule to interpret differences between means. The downside is that it is not immediately apparent which error bars belong to which pair of means. The researcher needs to decide whether compactness of presentation outweighs this limitation.

To create a plot like Fig. 1f, each *SEM* ^pairedDiff^ is multiplied by $\frac{1}{{\sqrt {2} }}$ and then plotted as an error bar for each of the two means from which the difference was calculated. The scaling is necessary because we go back from a difference of two means to two single means. The scaling gives us, for each mean, the *SEM* that would correspond to the *SEM* of the difference if the two means were independent and had the same variability, such that the 3-*SEM* rule can be applied and the scaled *SEM* ^pairedDiff^s are compatible with the *SEM* ^L&M^s (Appendix. A1).

## Generalization to multifactor experiments

**Fig. 4:**
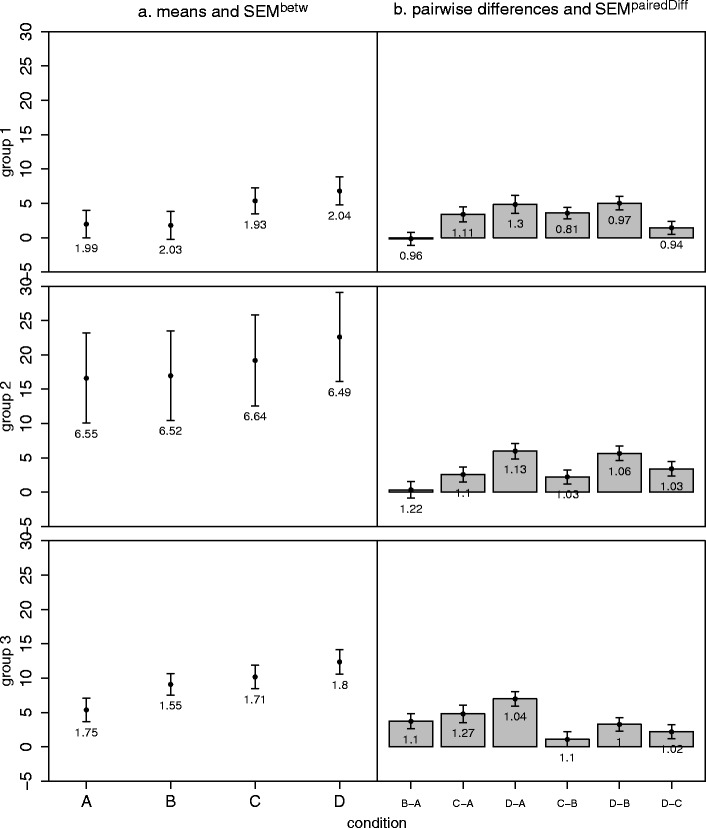
Generalization of our approach to mixed designs. The example has one between-subjects factor with three levels (Groups 1–3) and one within-subjects factor with four levels (conditions A–D) (**a**) Means and the corresponding *SEM* ^betw^ values. Group 2 has larger *SEM* ^betw^s, indicating a violation of the homogeneity assumption. (**b**) Pairwise differences and the corresponding *SEM* ^pairedDiff^s indicate no violation of circularity. Error bars depict ±1 *SEM*s as calculated by the different methods. Numbers below the error bars are the numerical values of the *SEM*s
  Inspecting Fig. 4a shows that Group 2 has higher *SEM* ^betw^s than the other groups, suggesting a violation of the HOV assumption. And indeed, the four corresponding Levene (1960) tests, each comparing the variability of the groups at one level of the within-subjects factor, show a significant deviation from HOV (all *F*s > 27, all *p*s < .001). Our approach reveals that this is due to the higher variability of Group 2. Inspecting Fig. 4b shows that the *SEM* ^pairedDiff^s are similar, suggesting that circularity is fullfilled. This, again, is consistent with standard repeated measures methods (Greenhouse–Geisser *ε* = .960, Huynh–Feldt *ε* = 1, Mauchly test *W* = .944, *p =* .25).

- ***Only within-subjects factors*** So far, we have discussed only single-factor designs. If more than one repeated measures factor is present, the *SEM* ^pairedDiff^ should be calculated across all possible pairwise differences. This simple method is consistent with the Loftus and Masson (1994) method, which also reduces multiple factors to a single factor (e.g., a 3 × 5 design is treated as a single-factor design with 15 levels).
  With regard to circularity, our generalization is slightly stricter than necessary, because we consider the pairwise differences of the variance–covariance matrix for the full comparison (by treating the design as a single-factor design). If the variance–covariance matrix fulfills circularity for this comparison, then it also fulfills it for all subcomparisons, but not vice versa (Rouanet & Lepine, 1970, Corollary 2). Therefore, it is conceivable that the *SEM* ^pairedDiff^ values indicate a violation of circularity, but that a specific subcomparison corresponding to one of the repeated measures factors does not. However, we think that the simplicity of our rule outweighs this minor limitation.
- ***Mixed designs (within- and between-subjects factors)*** In mixed designs, an additional complication arises because each group of subjects (i.e., each level of the between-subjects factors) has its own variance-covariance matrix, all of which are assumed to be homogeneous and circular. Thus, there are two assumptions, HOV and circularity. As was mentioned by Winer et al. (1991, p. 509), “these are, indeed, restrictive assumptions”—hence, even more need for a visual guide to evaluate their plausibility.
  Consider one within-subjects factor and one between-subjects factor, fully crossed, with equal group sizes. For each level of the between-subjects factor, we suggest a plot with the means and *SEM* ^betw^ for all levels of the within-subjects factor, along with a plot showing the pairwise differences and their *SEM* ^pairedDiff^ (Fig. 4 and Appendix A2). To evaluate the homogeneity and circularity assumptions, respectively, one would gauge whether all *SEM* ^betw^ values corresponding to the same level of the within-subjects factor were roughly equal and whether all possible *SEM* ^pairedDiff^s were roughly equal.

## Precautions

Although we believe our approach to be beneficial, it needs to be applied with caution (like any statistical procedure). Strictly speaking, the method only allows judgments about pairwise differences and the circularity assumption; it does not allow judgments of main effects or interactions. For this, we would need pooled error terms and overall averaging, as used in ANOVA. Also, our use of multiple estimates of variability (i.e., for each pairwise difference, a different *SEM* ^pairedDiff^) makes each individual *SEM* ^pairedDiff^ less reliable than an estimate based on the pooled error term. In many situations, however, neither restriction is a serious limitation.

For example, consider Fig. 1g. The *SEM* ^pairedDiff^ values are consistent, such that the *SEM* based on the pooled error term will be similar to them (Appendix A1) and that the inherently reduced reliability of the *SEM* ^pairedDiff^ will be no problem. Each pairwise difference suggests a significant difference from zero, be it interpreted as a-priori or post-hoc test,^7^ or by applying the 2-*SEM* rule of eye. Therefore, a reader seeing only this figure will have an indication that the main effect of the ANOVA is significant. This example again shows how our method can supplement (though not supplant) traditional numerical methods.

## Conclusions

We have suggested a simple method to conceptualize variability in repeated measures designs: Calculate the *SEM* ^pairedDiff^ of all pairwise differences, and plot them. The homogeneity of the *SEM* ^pairedDiff^ provides an assessment of circularity and is (unlike the normalization method) a valid generalization of the well-established Loftus and Masson (1994) method.

## Appendix

### **A1. Relationship between*****SEM***^**pairedDiff**^**and*****SEM***^**L&M**^

We show that the *SEM* ^L&M^ is equal to the pooled and scaled *SEM* ^pairedDiff^ in the following way:

$$ SE{M^{{L\&M}}} = \sqrt {{\overline {{{\left( {\frac{1}{{\sqrt {2} }}SE{M^{{pairedDiff}}}_{{..}}} \right)}^2}} }} $$

This notation is similar to that of Winer et al. (1991): The horizontal line and the two dots indicate that all corresponding *SEM* ^pairedDiff^s are pooled. For example, in Fig. 1g, the *SEM* ^pairedDiff^ values are 0.3333, 0.2906, and 0.4163, such that

$$ SEM^{{L\& M}} = {\sqrt {\frac{{{\left( {\frac{1} {{{\sqrt 2 }}}SEM^{{pairedDiff}} 12} \right)}^{2} + {\left( {\frac{1} {{{\sqrt 2 }}}SEM^{{pairedDiff}} 13} \right)}^{2} + {\left( {\frac{1} {{{\sqrt 2 }}}SEM^{{pairedDiff}} 23} \right)}^{2} }} {3}} } = {\sqrt {\frac{{0.2357^{2} + 0.2055^{2} + 0.2944^{2} }} {3} = 0.2480} } $$

For the proof, consider a factor with *J* = 3 levels first. For a single-factor repeated measures ANOVA, $MSE \,=\, \overline {va{r_{.}}} - \overline {co{v_{{..}}}}$ (Winer et al., 1991, p. 264). Because $SE{M^{{L\&M}}} = \sqrt {{\frac{{MSE}}{n}}}$, we obtain

$$ SE{M^{{L\&M^2}}} = \frac{{MSE}}{n} = \frac{{\overline {va{r_{.}}} - \overline {co{v_{{..}}}} }}{n} = \frac{{va{r_1} + va{r_2} + va{r_3} - co{v_{{12}}} - co{v_{{13}}} - co{v_{{23}}}}}{{3n}} $$

The *SEM* for the difference between levels *k* and *l* is $SEM_{{kl}}^{{pairedDiff}} = \sqrt {{\frac{{va{r_k} - 2co{v_{{kl}}} + va{r_l}}}{n}.}}$ Multiplying by ${1}/\sqrt {{2}}$ and pooling gives

$$ \begin{array}{*{20}c} {\overline{{{\left( {\frac{1} {{{\sqrt 2 }}}SEM^{{pairedDiff}} ..} \right)}^{2} }} = \frac{1} {3}{\left( {\frac{1} {2}SEM^{{pairedDiff^{2} }}_{{12}} + \frac{1} {2}SEM^{{pairedDiff^{2} }}_{{13}} + \frac{1} {2}SEM^{{pairedDiff^{2} }}_{{23}} } \right)}} \\ { = \frac{{\operatorname{var} _{1} - 2\operatorname{cov} _{{12}} + \operatorname{var} _{2} + \operatorname{var} _{1} - 2\operatorname{cov} _{{13}} + \operatorname{var} _{3} + \operatorname{var} _{2} - 2\operatorname{cov} _{{23}} + \operatorname{var} _{3} }} {{6n}}} \\ { = \frac{{\operatorname{var} _{1} + \operatorname{var} _{2} + \operatorname{var} _{3} - \operatorname{cov} _{{12}} - \operatorname{cov} _{{13}} - \operatorname{cov} _{{23}} }} {{3n}} = SEM^{{L\& M^{2} }} } \\ \end{array} $$

Generalization to a factor with more than three levels: There are *J*(*J* – 1)/2 pairwise differences, *J*(*J* – 1)/2 covariances, and *J* variances. This gives

$$ \matrix{{*{20}{c}} {\overline {{{\left( {\frac{1}{{\sqrt {2} }}SE{M^{{pairedDiff}}}_{{..}}} \right)}^2}} \quad = \quad \frac{2}{{J\left( {J - 1} \right)}}\sum\limits_{{K = 1}}^{{J1}} {\sum\limits_{{l = k + 1}}^J {\frac{1}{2}SEM_{{kl}}^{{pairedDiff2}}} } } \hfill \\ {\quad \quad \quad \quad \quad \quad \quad \quad \quad = \quad \quad \frac{2}{{J\left( {J - 1} \right)}}\frac{1}{{2n}}\sum\limits_{{k = 1}}^{{J - 1}} {\sum\limits_{{l = k + 1}}^J {va{r_k} - 2co{v_{{kl}}} + va{r_l}} } } \hfill \\ {\quad \quad \quad \quad \quad \quad \quad \quad \quad = \quad \quad \frac{1}{n}\left( {\frac{1}{{J\left( {J - 1} \right)}}\sum\limits_{{k = 1}}^J {\left( {J - 1} \right)va{r_k} - \frac{1}{{J\left( {J - 1} \right)}}\sum\limits_{{k = 1}}^{{J - 1}} {\sum\limits_{{l = k + 1}}^J {2\,co{v_{{kl}}}} } } } \right)} \hfill \\ {\quad \quad \quad \quad \quad \quad \quad \quad \quad = \frac{1}{n}\left( {\frac{1}{J}{{\sum\limits_{{k = 1}}^J {var}_k}} - \frac{2}{{J\left( {J - 1} \right)}}\sum\limits_{{k = 1}}^{{J - 1}} {{{\sum\limits_{{l = k + 1}}^J {cov}_{kl}}}} } \right)} \hfill \\ {\quad \quad \quad \quad \quad \quad \quad \quad \quad = \frac{1}{n}\left( {\overline {va{r_{.}} - } \,\overline {co{v_{{..}}}} } \right) = SE{M^{{L\&M2}}}} \hfill \\ } $$

### **A2. Mixed designs**

We treat all within- and between-subjects factors of a mixed design as single factors, such that we reduce the problem to one between- and one within-subjects factor. In such a two-factor mixed design, there is for each level of the between-subjects factor a different variance–covariance matrix for the within-subjects factor, which all have to be homogeneous and circular (Winer et al., 1991, p. 506). If group sizes are equal, this can be assessed in three steps: (a) Estimate for each level of the within-subjects factor, whether the corresponding *SEM* ^betw^ values are equal across all levels of the between-subjects factor. If this is the case, the entries on the diagonal of the variance–covariance matrices (i.e., the variances) are equal. (b) Estimate for each pair of within-subjects levels whether the corresponding *SEM* ^pairedDiff^ values are equal across all levels of the between-subjects factor. This ensures that all off-diagonal elements of the variance–covariance matrices (i.e., the covariances) are equal, because we already know that the variances are equal and, due to the relationship

$$ SEM_{{kl}}^{{pairedDiff}} = \sqrt {{\frac{{va{r_k} - 2co{v_{{kl}}} + {{{\rm var} }_l}}}{n}}}, $$

the *SEM*_*kl*_^pairedDiff^s can only be equal if the covariances are equal. (c) Estimate for each level of the between-subjects factor whether the *SEM* ^pairedDiff^s corresponding to all pairs of within-subjects levels are equal. This ensures the circularity of the variance–covariance matrices.

In short, we need to assess whether all *SEM* ^betw^ values at each level of the within-subjects factor are similar, and whether all *SEM* ^pairedDiff^s are similar. With unequal group sizes, we cannot use *SEM*, because a different *n* would enter the calculation. Therefore, we need to use standard deviations instead.

## Footnotes

1 For simplicity, the 3-*SEM* rule treats all comparisons as a-priori contrasts and does not take into account problems of multiple testing. Below we provide an example of Bonferroni correction for post-hoc testing. Similarly, one could calculate confidence intervals based on Tukey’s range test or similar statistics.

2 Note that the *SEM* ^L&M^ only provides information about the *differences* among within–subject levels. It does not provide information about the *absolute value* of the *DV*, for which *SEM* ^betw^ would be appropriate. It is, however, rare in psychology that absolute values are of interest.

3 Unfortunately, this illustration has led to some confusion. Although it provides a valid description of the error term in the repeated measures ANOVA, it suggests that the Loftus and Masson (1994) method was based on normalized scores, which is not true. Therefore, the normalization method is not a generalization of the Loftus and Masson method. Also, the critique based on the assumption that the Loftus and Masson method used normalized scores (Blouin & Riopelle, 2005) does not apply.

4 That the normalization method is biased might confuse some readers, because they remember that we can represent a within-subjects ANOVA as a between-subjects ANOVA on the normalized scores (Maxwell & Delaney, 2000, p. 472, note 5 of chap. 11). However, to obtain a correct *F* test, we would need to deviate from the between-subjects ANOVA by adjusting the degrees of freedom (Loftus & Loftus, 1988, digression 13-1, p. 426). This adjustment takes into account that the normalized data are correlated and is not performed by the normalization method.

5 The Bonferroni correction is this: We have six possible comparisons. Therefore, we need the (100 – 5/6)% = 99.12% criterion of the *t* distribution with (40 – 1) = 39 degrees of freedom, which is *t*_crit_ = 2.78. Therefore, all *SEM*s need to be multiplied by this value (instead of 2, as in the 2-SEM rule).

6 In our example, MANOVA is more appropriate because it does not rely on the assumption of circularity. It has, however, other limitations (mainly for small sample sizes) such that it cannot simply replace univariate ANOVA in general.

7 As an example, let us calculate the confidence interval (CI) for the difference “2 s–1 s”: (a) A-priori test: The 95% critical value of the *t* distribution is *t*_crit95%_(9) = 2.26, resulting in a CI of 2 ± (0.33 * 2.26) = [1.25, 2.75]. (b) Post-hoc test with Bonferroni correction: With *J* = 3 pairwise comparisons, we need the (100 – 5/3) = 98.33% criterion of the *t* distribution, which is *t*_crit98.33%_(9) = 2.93, and the CI is calculated as 2 ± (0.33*2.93) = [1.03, 2.97].

## References

[CR1] Bakeman R, McArthur D (1996). Picturing repeated measures: Comments on Loftus, Morrison, and others. Behavior Research Methods, Instruments, & Computers.

[CR2] Blouin DC, Riopelle AJ (2005). On confidence intervals for within-subjects designs. Psychological Methods.

[CR3] Box GEP (1954). Some theorems on quadratic form applied in the study of analysis of variance problems: II. Effects of inequality of variance and of correlation between errors in the two-way classification. Annals of Mathematical Statistics.

[CR4] Box GEP (1954). Some theorems on quadratic forms applied in the study of analysis of variance problems: I. effect of inequality of variance in the one-way classification. Annals of Mathematical Statistics.

[CR5] Cousineau D (2005). Confidence intervals in within-subject designs: A simpler solution to Loftus and Masson’s method. Tutorials in Quantitative Methods for Psychology.

[CR6] Cumming G, Finch S (2005). Inference by eye: Confidence intervals and how to read pictures of data. American Psychologist.

[CR7] Goldstein H, Healy MJR (1995). The graphical presentation of a collection of means. Journal of the Royal Statistical Society: Series A.

[CR8] Greenhouse SW, Geisser S (1959). On methods in the analysis of profile data. Psychometrika.

[CR9] Huynh H, Feldt LS (1976). Estimation of the Box correction for degrees of freedom from sample data in randomized block and split-plot designs. Journal of Educational Statistics.

[CR10] Huynh L, Feldt S (1970). Conditions under which mean square ratios in repeated measurements designs have exact *F*-distributions. Journal of the American Statistical Association.

[CR11] Levene H, Olkin I (1960). Robust tests for equality of variances. *Contributions to probability and statistics*.

[CR12] Loftus GR, Loftus EF (1988). *Essence of statistics*.

[CR13] Loftus GR, Masson MEJ (1994). Using confidence intervals in within-subject designs. Psychonomic Bulletin and Review.

[CR14] Mauchly JW (1940). Significance test for sphericity of a normal *n*-variate distribution. Annals of Mathematical Statistics.

[CR15] Maxwell SE, Delaney HD (2000). *Designing experiments and analyzing data: A model comparison perspective*.

[CR16] Morey RD (2008). Confidence intervals from normalized data: A correction to Cousineau (2005). Tutorials in Quantitative Methods for Psychology.

[CR17] Morrison GR, Weaver B (1995). Exactly how many *p* values is a picture worth? A commentary on Loftus’s plot-plus-error-bar approach. Behavior Research Methods, Instruments, & Computers.

[CR18] Rouanet H, Lepine D (1970). Comparison between treatments in a repeated-measurement design—ANOVA and multivariate methods. British Journal of Mathematical and Statistical Psychology.

[CR19] Tryon WW (2001). Evaluating statistical difference, equivalence, and indeterminacy using inferential confidence intervals: An integrated alternative method of conducting null hypothesis statistical tests. Psychological Methods.

[CR20] Winer BJ, Brown DR, Michels KM (1991). *Statistical principles in experimental design* (3.

